# Integrated AHP-Delphi and Mini-HTA for selection of metabolic drugs in China’s national medical insurance catalog: a framework towards quantitative dynamic adjustment

**DOI:** 10.1080/20523211.2026.2701914

**Published:** 2026-09-21

**Authors:** Jiajin Li, Tianxiao Huang, Yujie Sun, Lu Yu

**Affiliations:** a College of Future Technology, Peking University, Beijing, People’s Republic of China; b School of Future Technology, Xi'an Jiaotong University, Xi'an, People’s Republic of China; c Sinopharm Healthcare Medical Expert Committee, Sinopharm Group, Beijing, People’s Republic of China

**Keywords:** Metabolic medicines, China's national medical insurance catalog, mini-HTA, AHP-Delphi, dynamic quantitative selection​​

## Abstract

**Background:**

China’s high burden of metabolic disease requires more systematic approaches to selecting medicines for inclusion in the National Medical Insurance Catalog. Existing processes face difficulties in quantitatively balancing clinical value, target population needs, pharmacoeconomic evidence, and resource constraints, and may therefore rely heavily on subjective judgment.

**Methods:**

We developed a decision framework integrating the analytic hierarchy process (AHP), the Delphi method, and mini-health technology assessment (mini-HTA) principles. Candidate criteria were identified from the WHO Essential Medicines List, India’s National List of Essential Medicines, the Kenya Essential Medicines List, China’s National Medical Insurance Catalog, and relevant policy-oriented literature on drug selection in China. These criteria were used to construct a systematic and locally adapted framework. Three rounds of consultation were conducted with a multidisciplinary expert panel comprising one clinician, three pharmacists, and three health insurance administrators. Indicator weights were calculated using the geometric mean method. Expert agreement and judgment consistency were assessed using Kendall’s coefficient of concordance (W) and consistency ratios (CR), respectively.

**Results:**

The framework included 14 primary indicators and 42 secondary indicators. Expert consensus was statistically significant (Kendall’s W = 0.57, p < 0.001), and all consistency ratios were below 0.1, indicating acceptable consistency and robustness in weight allocation.

**Conclusions:**

The proposed AHP–Delphi–mini-HTA framework provides a quantitative and transparent approach to selecting metabolic drugs for insurance coverage in China. Although prospective pilot testing is still required, the framework supports standardized assessment, reduces reliance on subjective judgment, and may facilitate future integration of real-world evidence and AI-assisted decision-making.

## Background

Metabolic diseases pose a severe public health crisis in China, characterised by high prevalence and clinical severity. According to the International Diabetes Federation (IDF) Diabetes Atlas released in 2025, China had approximately 148 million adults aged 20–79 years living with diabetes by 2024, representing the world's largest diabetic population, the nation's total health expenditure attributable to diabetes ranked second-highest globally (International Diabetes Federation, 2025). Concurrently, over 50% of adults are overweight/obese, a condition strongly associated with chronic multimorbidity and premature mortality (National Health Commission of the PRC, 2020). Hypertension affects 27.5% of the population, yet demonstrates critically low awareness (51.6%), treatment (45.8%), and control rates (16.8%), directly contributing to elevated risks of cardiovascular events and end-stage renal disease (b1 Writing Group of Chinese Guidelines for the Management of Hypertension, 2024). Alarmingly, elevated systolic blood pressure (SBP) alone caused 2.54 million deaths in 2017, underscoring the urgency for optimised therapeutic interventions (National Health Commission of the People’s Republic of China, 2023).

Pharmacotherapy remains pivotal for managing metabolic diseases, yet economic accessibility critically determines intervention efficacy. Take diabetes: metformin monotherapy costs US$4–100/month, while multidrug regimens escalate to US$200–500/month – prohibitively expensive for most households (CostHelper Health). China’s national health insurance has mitigated this through a 95% price reduction policy (Social Security Network, 2025), with the National Medical Insurance Catalog incorporating multiple metabolic therapies to enable sustained treatment adherence(Mao et al., 2019). However, a comprehensive analysis of the currently released policy documents from the National Healthcare Security Administration (NHSA) and the research literature on metabolic drugs reveals that three major systemic challenges still urgently need to be addressed (China News Service, 2024): (1) Pharmacological heterogeneity – metabolic agents exhibit marked variations in efficacy, safety,and target populations (Fuchigami et al., 2020; Li et al., 2023; Ye et al., 2021), complicating quantitative prioritisation via conventional expert panels; (2) Funding efficiency dilemmas**⁣**⁣ – balancing pharmacoeconomic metrics against clinical value under constrained resources (General Office of the State Council of the People's Republic of China, 2023); (3) Transparency deficits – the absence of standardised evaluation frameworks perpetuates over-reliance on subjective judgments, undermining evidence-based decision-making.

Within this context, health technology assessment (HTA) emerges as a pivotal evidence-based policy tool, offering structured frameworks to optimise drug selection. International evidence demonstrates HTA’s capacity to enhance healthcare resource allocation through multidimensional evidence synthesis – clinical efficacy, cost-effectiveness and ethical considerations (Li Jiajin et al., 2024; O'Rourke et al., 2020). However, traditional HTA’s resource-intensive nature conflicts with China’s urgent need for dynamic adjustment of the National Medical Insurance Catalog. Mini-HTA addresses this gap by simplifying evaluation criteria and processes, as evidenced by its successful integration into a quick guideline for drug evaluation and selection in chinese medical institutions (the second edition) (Zhao Zhigang et al., 2023). Despite this progress, mini-HTA’s potential remains underutilised in national reimbursement decision-making systems, particularly for metabolic therapeutics.

This study innovatively integrates the Analytic Hierarchy Process-Delphi (AHP-Delphi) method with a mini-HTA framework to systematically develop a metabolic drug selection index system. The AHP methodology, pioneered by Saaty in the 1970s (Saaty, 1977), quantifies complex decision-making through four stages: hierarchical modelling, pairwise comparison matrices, weight calculation and consistency checks (CR < 0.1), effectively reconciling subjective-objective data conflicts (Saaty, 1987). Its proven efficacy in healthcare prioritisation (Dolan et al., 1989; Liberatore & Nydick, 2008; Vaidya & Sushil, 2006) motivated our adoption, particularly due to three strengths: (1) Hierarchical resolvability ⁣⁣ – decoding multidimensional indicator conflicts via tiered weight analysis, (2) Consensus convergence ⁣⁣ – bridging policymakers’ weighting discrepancies through CR validation and (3) Group compatibility **⁣**⁣ – geometric mean aggregation rules harmonising Delphi expert inputs while minimising outlier biases. Although the AHP-Delphi method has been systematically applied in multiple fields, such as healthcare security research and practice – as exemplified by the article ‘Study on the Construction and Application of Evaluation Index System Assessing Quality of Medical Insurance Service Based on DIP with Delphi Method and AHP’ (Teng Jiali et al., 2023) published in the Peking University core journal China Health Policy Research, which utilised two rounds of Delphi expert consultation to establish the evaluation indicator system and employed AHP to determine the relative weights of indicators at all levels – the integration of the AHP-Delphi method into a mini-HTA-based framework for metabolic drug selection and its systematic construction tailored to the decision-making context of dynamic adjustment in China's medical insurance catalog still represents an exploratory methodological innovation. Leveraging systematic evidence synthesis, this study integrated metabolic drug selection criteria from the WHO EML, NLEM of India(Anjana et al., 2023; Geldsetzer et al., 2018) and KEML (Kenya National Bureau of Statistics, 2022; Temu et al., 2021) (selected for their analogous high metabolic disease burdens under resource constraints), and China-specific indicators from both National Medical Insurance Catalog and high-impact studies on health insurance systems and healthcare institutional criteria indexed in China National Knowledge Infrastructure (CNKI), WanFang Data and VIP Information (VIP). Unlike the single-catalog model used by the World Health Organisation and most countries, China's system features a substantive functional division: the NEDL ensures drug accessibility by guiding essential medicine supply in medical institutions, especially primary care facilities, while the National Medical Insurance Catalog enhances drug affordability by defining reimbursement scope through medical insurance funds. Notably, China's National Medical Insurance Catalog covers substantially more drugs (3,088 in the 2024 edition) than the NEDL (685 in the 2018 edition), with most essential drugs included in the catalog and typically receiving higher reimbursement rates. Thus, in achieving the dual objectives of ‘guaranteeing essential medicine access' and ‘cost reimbursement,' China's National Medical Insurance Catalog functionally aligns more closely with conventional international essential medicines lists. CNKI, Wanfang Data, and VIP are the three dominant academic databases in China, collectively forming the core infrastructure for retrieving and accessing Chinese scholarly literature. Among them, CNKI , established by Tsinghua University and Tsinghua Tongfang in 1999, is the most comprehensive and highly recognised platform. It encompasses a wide range of document types, including journals, doctoral and master's theses, conference proceedings, newspapers, yearbooks, patents and standards, with a total literature volume exceeding 300 million entries. It holds a particularly significant advantage in the field of social sciences. Wanfang Data are authoritative in scientific and technological information, with a focus on science, engineering, agriculture and medicine, especially medical journals, as well as specialised resources like technical reports, patents, and standards. VIP Information is distinguished by having the most extensive collection of journal titles (approximately 15,000), with its archival literature dating back to 1955, and offers broad coverage in natural sciences and engineering technology journals. The analysis focuses on China's National Medical Insurance Catalog rather than the National Essential Drugs List (NEDL) due to its distinctive ‘dual-catalog' structure.

In this context, this study aims to construct a rapid decision-making framework applicable to medical insurance reimbursement policies for metabolic drugs, to support dynamic quantitative assessment and provide a reference paradigm for regions with imbalanced resource allocation.

## Methods

### Research implementation framework

The overall research methodology was implemented through the structured approach summarised in Table 1.

**Table 1.** ⁣**⁣** Research methodology implementation framework⁣⁣.

⁣⁣PHASE	⁣⁣STAGE
⁣⁣Evidence synthesis⁣⁣	International policy frameworks
	Chinese institutional guidelines
	Mini-HTA
⁣⁣Literature screening⁣⁣	Independent Screening
	Inclusion/exclusion criteria
⁣⁣AHP process⁣⁣	Hierarchical construction
	Judgment matrix development
⁣⁣Delphi process⁣⁣	Questionnaire design
	Iterative rounds
⁣⁣Data synthesis & weighting⁣⁣	Geometric mean aggregation
	Weight derivation
	Consistency validation
**Repeated the aforementioned steps** for secondary indicators under each primary category, then multiplied the subgroup weights by their parent weights for global prioritisation.	
⁣**⁣Final Output⁣**⁣: A finalised, weighted indicator framework for metabolic drug selection and prioritisation.	

Abbreviations: AH*P* = analytic hierarchy process; mini-HTA = mini-health technology assessment.

### Data sources and searching strategies

This study synthesised multi-source evidence through various methods:

1. International policy frameworks:

Metabolic drug selection criteria from WHO EML (World Health Organisation, 2023b), KEML (World Health Organisation, 2023a) and India’s NLEM (Ministry of Health and Family Welfare, 2022), selected for their validated adaptation to resource-constrained settings (Yan Jianzhou et al., 2022);

(2) Chinese institutional guidelines:

Metabolic drug selection criteria from the China National Medical Insurance Catalog. Systematic searches across CNKI, WanFang and VIP databases (1 June 2020–1 June 2025), supplemented by official announcements from the National Health Commission and Healthcare Security Administration. Search strategies combined the formula ‘SU = (‘National Reimbursement Drug List' | ‘National Medical Insurance Catalog') AND ABS = (‘indicator*' | ‘evaluation*’ | ‘framework*' | ‘assess*' | ‘select*' | ‘criteria') AND YE > 2019’ with document filters (academic journals, dissertations, policy white papers).

(3) Mini-HTA:

Mini-HTA provides a streamlined and efficient core dimension evaluation framework as a foundational basis for underlying design. Building upon this framework, and targeting the selection needs of metabolic drugs, it systematically references and integrates the aforementioned authoritative international and domestic policy reports along with high-level Chinese scientific literature to conduct targeted expansion and adaptation of this framework.

### Literature inclusion and exclusion criteria

Two researchers independently executed literature screening per predefined protocols, utilising EndNote 21 for deduplication, with discrepancies resolved by a senior investigator (Kappa statistic = 0.87). Inclusion criteria encompassed: (1) peer-reviewed Chinese publications (1 June 2020–1 June 2025) reporting measurable indicators/weights for China's National Medical Insurance Catalog selection; (2) drug evaluation guidelines from authoritative healthcare institutions and (3) official announcements from NHSA. Exclusion criteria eliminated duplicate publications, conference abstracts, study protocols and incomplete reports.

### AHP-Delphi process

The AHP was operationalised through: (1) ⁣Hierarchical construction: Establishing a tree-structured decision hierarchy with 14 Primary Indicators and 42 Secondary Indicators derived from systematic evidence extraction; (2) ⁣⁣Judgment matrix development⁣⁣: 7 experts performed pairwise comparisons using Saaty's 1–9 scale (as shown in Table 2), generating reciprocal judgment matrices$\;A^{\left(k\right)}=$[$a_{\mathrm{ij}}^{\left(k\right)}$] that satisfy$\;a_{\;\mathrm{ji}}^{\left(k\right)}=\frac{1}{a_{\mathrm{ij}}^{\left(k\right)}}$, $a_{\mathrm{ii}}^{\left(k\right)}=1$ for transitivity consistency.

**Table 2.** Saaty 1–9 scale scoring.

Value	Interpretation
1	Equal importance between indicators
3	Indicator iis slightly more important than indicator j
5	Indicator iis strongly more important than indicator j
7	Indicator iis very strongly more important than indicator j
9	Indicator iis absolutely more important than indicator j
2, 4, 6, 8	Intermediate values representing compromises between adjacent levels

Note. This table serves as a conceptual reference framework for the pairwise comparison standard utilised in the AHP methodology.

Regarding the determination of the expert sample size, classical methodological literature emphasises that the validity of Delphi and AHP techniques relies on ‘domain authority' and ‘heterogeneity of perspectives' rather than the large-sample laws typical of traditional epidemiological surveys. When an expert panel possesses high-level expertise in a specific niche, a relatively small sample size is sufficient to ensure statistical stability and achieve ‘theoretical saturation’, whereas arbitrarily expanding the panel with non-core experts may instead lead to opinion dilution and noise interference (Akins et al., 2005; Guest et al., 2006). Prioritising depth of domain expertise over broad numerical representation in accordance with these principles, the Delphi iteration in this study engaged a targeted panel of 7 experts (clinician: n = 1; pharmacists: n = 3; health insurance administration staff: n = 3; all possessing ≥5 years’ experience in metabolic therapeutic policy-making) through three-round structured consultations. Customised questionnaires comprised: (1) a cover page with informed consent detailing research protocols/risks/privacy safeguards; (2) a demographic section (name, gender, age, professional title, expertise, etc.) and (3) hierarchical comparison modules – primary indicators (14 indicators, 91 pairwise comparisons) and secondary subgroup evaluations (42 indicators clustered under respective primary categories).

The Delphi iteration for primary indicators comprised three phases: (1) Round 1: Open-ended questionnaire distribution to collect initial expert judgments; (2) Round 2: Anonymous feedback of AHP-derived weights (via geometric mean aggregation) with mandatory rationale documentation for revised responses and (3) Termination criteria: Consensus achievement or completion of a maximum of 3 rounds. A Kendall’s concordance coefficient (W) ≥ 0.5 was set as the threshold for consensus. According to standard statistical interpretations for Delphi studies, a W value between 0.5 and 0.7 indicates a moderate, acceptable level of expert agreement, confirming sufficient convergence to terminate the iterations.

The geometric mean aggregation method was applied to synthesise expert inputs, minimising outlier impacts via: (1) Expert matrix synthesis: For m experts, calculate each element $g_{\mathrm{ij}}=\sqrt[{m}]{a_{\mathrm{ij}}^{\left(1\right)}\times a_{\mathrm{ij}}^{\left(2\right)}\times \cdots \times a_{\mathrm{ij}}^{\left(m\right)}}$to construct the group judgment matrix$\;G=[g_{\mathrm{ij}}{]}_{n\times n}$⁣; (2) Weight derivation: Compute row geometric means $r_{i}=\sqrt[{n}]{g_{i1}\times g_{i2}\times \cdots \times g_{\mathrm{in}}}$, then normalise to obtain global weights $W_{i}=\frac{r_{i}}{\sum_{i=1}^{n}r_{i}}$; (3) Consistency validation: Verify $\mathrm{CR}=\frac{\mathrm{CI}}{\mathrm{RI}}<0.1\;(\mathrm{threshold}\;\mathrm{per}\;\mathrm{Saaty}),\;\;\mathrm{where}$ $\mathrm{CI}=\frac{\lambda_{\max}-n}{n-1},\;\lambda_{\max}=\frac{1}{n}\sum_{i=1}^{n}\frac{{\left(\mathrm{AW}\right)}_{i}}{W_{i}}$), and *RI* values referenced from standard tables; (4) Hierarchical synthesis: Repeat steps 1–3 for secondary indicators under each primary category, then multiply subgroup weights by parent weights for global prioritisation.

## Results

Based on the mini-HTA framework, this study expanded and tailored the framework to better align with the specificities of metabolic drug selection in China by integrating multi-source evidence. The newly established hierarchical evaluation system comprises 14 primary indicators and 42 secondary indicators. Using a hybrid AHP-Delphi method, weights were assigned to each indicator to enable quantitative analysis. Through three rounds of iterative consultations with a multidisciplinary expert panel (n = 7), a moderate level of consensus was achieved (Kendall’s W = 0.57, *p* < 0.001). All consistency ratios (CR < 0.1) confirmed the logical coherence and robustness of the judgment matrices. The detailed findings are presented in the following sections: [Results of Literature Search], [Development of a Multidimensional Evaluation Framework for Metabolic Drugs] and [Questionnaire Survey and Statistical Analysis].

### Results of literature search

This study systematically synthesizes relevant policy documents and academic evidence regarding medication selection criteria for China's national medical insurance catalog: (1) Authoritative policy frameworks issued by the NHSA: (a) The *Interim Measures for the Administration of Medicines Covered by Basic Medical Insurance * (National Healthcare Security Administration (NHSA), 2020) systematically establishes the governing criteria for medicine selection under the medical insurance formulary. It covers key aspects such as inclusion, exclusion, dynamic adjustment and payment management . The explicit core criteria for formulary inclusion are summarised as follows: ‘Clinically essential, safe and effective, reasonably priced, user-friendly, stably supplied, and compliant with the statutory approval standards of the National Medical Products Administration (NMPA).' (b) The *Work Plan for the Adjustment of the National Basic Medical Insurance, Workers’ Compensation, and Maternity Insurance Medicine Catalogue (2024)* ((NHSA), 2024) establishes a multidimensional evaluation framework, encompassing fundamental drug attributes, safety, efficacy, and other critical dimensions for formulary selection. (2) Academic synthesis : (a) A systematic search was conducted across CNKI, Wanfang , and VIP databases , yielding 141 records (CNKI = 54, Wanfang = 58, VI*P* = 29). (b) Duplicate records were removed using ⁣⁣EndNote⁣⁣, followed by title/abstract screening; 9 studies were retained for full-text review. (c) Full-text review under PICOS criteria (population: policies and regulations related to the national medical insurance catalog; intervention/indicator: indicator systems, evaluation models, or frameworks for national medical insurance catalog drug selection; context: China’s healthcare systems; outcome: Construction, application, or optimisation proposals for indicator systems; Study design: Policy analysis literature) identified 3 highly relevant studies , including: a multi-dimensional decision framework for health technology assessment in national medical insurance catalog adjustments (Wang Ting, 2022), medical insurance drug admission: (colon)a comparison of mini – HTA and RHTA (Yunxiang et al., 2025) and a study on the multidimensional decision framework for health technology assessment from the perspective of adjusting the medical insurance drug catalog (Yang Yan et al., 2023). (d) To ensure ⁣⁣comprehensive indicatorcoverage , two authoritative guidelines on hospital drug selection models were added: Drug Selection Guideline for Medical Institutions (Zhengxiang et al., 2022), and A Quick Guideline for Drug Evaluation and Selection in Chinese Medical Institutions (the Second Edition; Zhigang et al., 2023).

### Development of a multidimensional evaluation framework for metabolic drugs⁣

The developed metabolic drug selection framework comprises 14 Primary Indicators and 42 Secondary Indicators, systematically covering critical therapeutic evaluation criteria (Supplementary Table S1).

Dimensions 1.1 (public health relevance of diseases) and 2.1–2.4 (clinical essentiality of medicines) prioritise disease-drug alignment; 3.1–3.2 (prescribing information) and 4.1–4.7 (clinical effectiveness of medicines) assess evidence strength and clinical boundaries; 5.1–5.2 (pharmacokinetic profiles) and 6.1–6.5 (safety of medicines) evaluate metabolic pathways and adverse reaction risks.

To address chronic medication needs in metabolic diseases, the framework specifically incorporates long-term management indicators: 7.1–7.5 (quality of medicines), 8.1–8.2 (cost-effectiveness of medicines), 10.1–10.6 (ease of administration and MEDICATION adherence), 11.1–11.2 (accessibility of medicines) and 12.1 (special requirements of Medicines).

The framework integrates policy adaptability dimensions, including value assessment indicators such as 13.1 (equity in access to medicines) and 14.1–14.3 (innovation in medicines), forming a multidimensional evaluation matrix that balances technical attributes and policy orientation, thereby ensuring scientific rigour and regulatory compliance in drug selection decisions.

### Questionnaire survey and statistical analysis

Employing an AHP-Delphi methodology, this study implemented three iterative consultation rounds with 8 distributed questionnaires per phase, achieving an 87.5% response rate (7/8 valid responses). The multidisciplinary panel (male:5 [71.4%], female: 2 [28.6%]; clinicians/pharmacists: 4 [57.1%], healthcare policymakers:3 [42.9%]; 100% bachelor's degree or higher; professional experience: 11–20 years [71.4%], 6–10 years [14.3%], >20 years [14.3%]) demonstrated robust engagement, with response durations distributed as follows: Questionnaire 1–500–1000 s (85.7%), ≥1001 s (14.3%); Questionnaire 2–500–1000 s (57.1%), ≤500 s (28.6%), ≥1001 s (14.3%), as shown in Table 3.

**Table 3.** Expert basic characteristics.

Basic characteristics	Number of participants (percentage)
**Gender**	
Male	5 (71.4%)
Female	2 (28.6%)
**Occupation**	
Clinical specialist	1 (14.3%)
Pharmacists	3 (42.9%)
Health insurance policy analyst	3 (42.9%)
**Educational background**	
Bachelor's degree or higher	7 (100%)
other	0 (0%)
**Years of professional experience**	
≤ 5 (Years)	0 (0%)
6–10 (Years)	1 (14.3%)
11–20 (Years)	5 (71.4%)
≥ 21 (Years)	1 (14.3%)
**Response time (Questionnaire 1)**	
≤ 500 (Seconds)	0 (0%)
501–1000 (Seconds)	6 (85.7%)
≥ 1001 (Seconds)	1 (14.3%)
**Response time (Questionnaire 2)⁣⁣**	
≤ 500 (Seconds)	2 (28.6%)
501–1000 (Seconds)	4 (57.1%)
≥ 1001 (Seconds)	1 (14.3%)

After two Delphi iterations, Kendall’s $W=0.57\;(\chi {\;}^{2}=51.87,\;\mathrm{df}=13,\;p<1.0\times 10^{-6})$ exceeding the 0.5 threshold, with $\chi {\;}^{2}=51.87$>critical value 22.362 (α=0.05,df=13), confirming moderate consensus (p<0.001) on 14 indicators..

The system of weights for each indicator calculated by geometric mean is presented in the paradigm of mini-HTA (as shown in Table 4).

**Table 4.** Hierarchical structure of metabolic drug selection framework derived from AHP and Delphi integration. Abbreviations: FDC = fixed-dose combination.

⁣Primary indicators (weighting)	Secondary indicators	Weighting⁣
1. Public health relevance of diseases (8.90%)	1.1 The epidemiological information of this disease in China (including total affected population, susceptible populations, prevalence rate, mortality rate, comorbidity incidence rate and others)	8.90%
2. Clinical essentiality of medicines (8.90%)	2.1 Medicines for rare diseases	2.10%
	2.2 Use of medicines in specific populations	2.66%
	2.3 Historical data on medication use	1.90%
	2.4 Limitations of existing medicines in clinical therapeutic use	2.24%
3. Prescribing Information (7.90%)	3.1 Target population, dosing regimen, treatment duration, adverse drug reactions, and pharmacokinetics	4.78%
	3.2 Is the completeness of additional information (pharmaceutical properties, drug interactions, drug overdose, specifications, and related parameters)	3.12%
4. Clinical effectiveness of medicines (8.77%)	4.1 Average score of inclusion status across pharmacopoeias: score for chinese pharmacopoeia inclusion, score for non-pharmacopoeial national drug standards inclusion, score for international pharmacopoeia inclusion frequency	0.87%
	4.2 Guideline recommendations: chinese mainland guideline recommendation score; southeast asian region guideline recommendation frequency score; other high-prevalence regions for metabolic diseases guideline recommendation frequency score⁣	1.02%
	4.3 Evidence-based medicine research findings: comparative impact of pharmacotherapy versus no intervention, versus comparator drugs, versus other therapeutic modalities on patient clinical outcomes: systematic review/meta-analysis (network) ranking score; randomized controlled trials ranking score; cohort studies or case-control studies ranking score; pathological reports or case observation studies ranking score⁣	1.60%
	4.4 Is the inclusion of indication-related diseases in clinical pathways⁣	1.19%
	⁣4.5 Is it an FDC⁣	1.11%
	4.6 Healthcare professionals’ high acceptance level⁣	0.78%
	4.7 Broad applicability: substantial evidence Demonstrates stable therapeutic efficacy in various settings (e.g. different healthcare levels)⁣	2.20%
5. Pharmacokinetic profiles (5.54%)	5.1 Are the absorption, distribution, metabolism, Excretion, and bioavailability of the medicine s⁣atisfactory⁣	3.25%
	⁣5.2 Is it nonlinear pharmacokinetics⁣	2.29%
6. Safety of medicines (14.31%)	6.1 Incidence of adverse reactions, severity (impact on patient quality of life, disease prognosis, and economic burden)⁣	2.58%
	6.2 Comparison of Adverse Reactions with Comparator Drugs	1.60%
	6.3 Reporting frequency of adverse reaction information (product labelling warnings and safety risk notifications/alerts/communications in China, US, Europe, and Japan)⁣	1.64%
	6.4 Contraindications and drug interactions⁣	2.36%
	6.5 Development status of the pharmacovigilance system⁣	1.84%
	6.6 ⁣Evidence and quality levels of safety studies for candidate medicines: systematic reviews and meta-analyses; randomized controlled trials; other types of clinical controlled studies⁣	1.93%
	6.7 Applicability of candidate medicines in special populations⁣	2.36%
7. Quality of medicines (15.84%)	7.1 Quality status scores of manufacturing enterprises⁣	2.75%
	7.2 Whether the drug is an innovator drug; if generic drugs have passed pharmaceutical equivalence evaluation⁣	3.54%
	7.3 ⁣Export and sales status of domestic drugs (registration, application, and sales status in foreign countries)⁣⁣	1.60%
	7.4 Development of quality standards control system⁣	3.71%
	7.5 Quality stability: compliance with internationally recognised quality standards acknowledged by pharmaceutical regulatory authorities, including in production, storage, transportation, and use⁣	4.24%
8. Cost-effectiveness of medicines (4.09%)	8.1 Cost-effectiveness comparisons with comparator drugs or other therapeutic modalities: unit price; daily cost; full course cost⁣	2.46%
	8.2 Cost-effectiveness literature research findings ranking score⁣	1.63%
9. Sales volume of medicines (1.89%)	9.1 Sales volume of candidate medicines⁣	1.89%
10. Ease of administration and medication adherence (5.75%)	10.1 Frequency of medication use⁣	0.74%
	10.2 Appropriate packaging, facilitated Accessibility, and adaptability to healthcare Institutions of all scales⁣	1.38%
	10.3 Route of administration (oral, injection, topical, etc.)⁣	1.10%
	10.4 Whether the taste of medication, side effects, etc. Align with patient preferences and needs⁣	0.69%
	10.5 Storage conditions of medicines⁣	0.86%
	10.6 Other information on adherence-related factors of candidate medicines	0.98%
11. Accessibility of Medicines (6.38%)	11.1 Marketing status of medicines; marketing status of generic drugs; whether the product has been approved by local pharmaceutical regulatory authorities⁣	2.22%
	11.2 Availability of candidate medicines (manifested by shortage level)⁣	4.16%
12. Special requirements of medicines 3.43%)	12.1 Whether the Use of the Product requires Professional Training, Diagnosis, Management, Monitoring, or Other Skills	3.43%
13. Equity in access to medicines (5.07%)	13.1 Whether inclusion would lead to diversion of limited healthcare funds from a larger patient population or higher-priority public health products; whether it pertains to a high-cost medicine with a small patient population, where non-inclusion may result in increased long-term treatment or care c⁣osts⁣	5.07%
14. Innovation in medicines (3.23%)	14.1 Whether candidate medicines can address gaps in the therapeutic formulary⁣	0.87%
	14.2 Whether candidate medicines demonstrate innovativeness in the scope of therapeutic indications⁣	1.22%
	14.3 Whether candidate medicines demonstrate innovativeness in the context of pharmacological mechanisms and pharmaceutical formulations⁣	1.14%

Abbreviations: FDC = Fixed-dose combination.

Geometric mean-weighted results showed CI = 0.03586 and CR = 0.02269 for primary indicators; all secondary indicators’ CIs/CRs were <0.1 (as shown in Table 5). This demonstrates a moderate, yet statistically significant, degree of expert consensus on the criticality of these indicators, establishing a stable and methodologically robust decision-making foundation for evidence-based drug selection.

**Table 5.** CI/CR values verify secondary indicator weights across primary dimensions.

	CI	CR
Public health relevance of diseases	-	-
Clinical essentiality of medicines	0.034142	0.037935
Prescribing information	-	-
Clinical effectiveness of medicines	0.01613	0.012217
Pharmacokinetic profiles	-	-
Safety of medicines	0.01899	0.01439
Quality of medicines	0.03240	0.02893
Cost-effectiveness of medicines	-	-
Sales volume of medicines	-	-
Ease of administration and medication Adherence	0.00957	0.00772
Accessibility of medicines	-	-
Special requirements of medicines	-	-
Equity in access to medicines	-	-
Innovation in medicines	0.00054	0.00094

Note. This table presents the consistency check results for the weight allocation of secondary indicators across 14 primary indicator dimensions. As per the AHP methodology, CI/CR values are calculated only when the number of secondary indicators within a group exceeds two, to validate the logical consistency of the judgment matrices. For groups containing only one or two secondary indicators, CI/CR values are omitted, and thus the corresponding cells are intentionally left blank. This is because such cases either cannot construct valid judgment matrices (for a single indicator) or naturally satisfy consistency conditions without requiring validation (for two indicators). A consistency ratio (CR) < 0.10 indicates acceptable consistency. Abbreviations: CI = consistency index; CR = consistency ratio.

In summary, the final weight distribution demonstrates the framework's internal consistency and its theoretical applicability for metabolic drug selection. The substantial weighting assigned to quality of medicines (15.84%), safety of medicines (14.31%) and clinical essentiality of medicines (8.90%) reflects a rational alignment between the experts’ moderate consensus (Kendall's W = 0.57) and the real-world demands of lifelong metabolic disease management. Methodologically, the framework at its current stage is primarily descriptive, as it quantitatively maps how multidisciplinary experts prioritise evaluation criteria under existing medical insurance constraints. However, its intended future application is prescriptive. By serving as a standardised quantitative tool, it aims to establish a transparent decision rule to guide the dynamic inclusion of metabolic therapeutics in the NMIC.

## Discussion

This instrument provides a standardised evaluation framework that enhances procedural standardisation and transparency in drug selection. Through weighted allocation across multiple dimensions such as safety, quality and clinical necessity, it enables more scientific drug evaluation, thereby assisting healthcare payers in both rationally adjusting the national medical insurance catalog and optimising formulary structures within medical institutions.

The weight distribution of selection criteria in this framework systematically reflects the chronicity, high prevalence, long-term medication requirements, and complication complexity of metabolic diseases. Safety of medicines (14.31%) and quality of medicines (15.84%) collectively account for over 30% of the primary indicators. While this might initially appear as an expert bias towards clinical endpoints, it actually perfectly aligns with the policy priorities of China's NMIC. Metabolic diseases require lifelong pharmacological management. Any instability in drug quality or severe adverse reactions not only directly threatens patient safety but also triggers severe complications, leading to a subsequent surge in medical insurance fund expenditures. Under China's ‘guaranteeing basic healthcare’ mandate, preventing systemic toxicity risks and ensuring long-term manufacturing quality are prioritised over other attributes. Therefore, this prominent influence is a rational reflection of NMIC’s macro-level fund security requirements rather than a mere clinical bias. Heightened weights for secondary indicators – such as incidence of adverse reactions (2.58%), contraindications and drug interactions (2.36%) and quality stability (4.24%) – further operationalise this mandate, directly addressing the elevated treatment risks in vulnerable populations. Public health relevance of diseases (8.90%) and clinical essentiality of medicines (8.90%) receive substantial weighting, reflecting metabolic diseases’ massive disease burden and therapeutic gaps requiring prioritised coverage. Furthermore, the secondary indicator of availability of candidate medicines (4.16%) under the primary accessibility of medicines dimension (6.38%), alongside the prominent weighting of ease of administration and medication adherence (5.75%), collectively forms dual safeguard mechanisms for long-term management. Notably, experts demonstrated extraordinary consensus (consistency index [CI] = 0.00054, consistency ratio [CR] = 0.00094) on assigning low priority to innovation in medicines (3.23%), reflecting the current therapeutic paradigm that prioritises the optimisation of existing therapies and risk-benefit equilibrium over short-term innovative breakthroughs. Overall, the weight distribution establishes a core logic centred on patient safety, therapeutic sustainability and public health imperatives, while balancing efficacy with user-friendly administration.

The marginalisation trend of innovation indicators also exposes the current system's insufficient incentives for the research and development (R&D) motivation of originator pharmaceutical enterprises. However, some metabolic diseases still lack specific therapeutic drugs, urgently requiring optimisation of innovation indicators to encourage the development of targeted therapies. Additionally, although innovative drugs have higher initial costs, they may reduce long-term medical expenditures by decreasing complications while improving patients’ quality of life. Encouragingly, with the deepening reform of drug review and approval systems, China's pharmaceutical innovation vitality has gradually been stimulated, providing institutional support to address these issues. In 2024, the total number of innovative drugs approved by China's National Medical Products Administration reached 48 varieties, a 20% year-on-year increase compared to 2023 and a significant 128.6% growth versus 2022, including 5 metabolic disease-specific drugs (Centre for Drug Evaluation, 2022, 2023, 2024). However, innovative pharmaceutical companies still face a prolonged investment return cycle (average 8–12 years) and funding chain disruption risks in R&D, leading to only a small fraction of enterprises being able to sustain their drug development pipelines. Against this backdrop, the state council general office issued the opinions on comprehensively deepening drug and medical device regulatory reforms to promote high-quality development of the pharmaceutical industry on 3 January 2025, proposing improved review and approval mechanisms to fully support major innovations and create transparent, stable, and predictable policy environments (General Office of the State Council of the People's Republic of China, 2024). It is noteworthy that the current drug R&D paradigm is undergoing profound transformation. International cutting-edge exploration indicates that new technologies like artificial intelligence have the potential to reshape the R&D process. For instance, PandaOmics(Kamya et al., 2024) integrated multi-omics data with causal inference models to identify TNIK – a novel target for idiopathic pulmonary fibrosis – and leveraged the/ generative chemistry platform Chemistry42 to design the inhibitor rentosertib. This end-to-end process, from target discovery to preclinical candidate nomination, was completed in just 18 months (Xu et al., 2025), this provides a potential new pathway to break through the dilemma of traditional long R&D cycles and high costs. The large-scale metabolic disease biobank (M-Biobank) constructed by the China Metabolic Analytics Project (ChinaMAP), which covers 27 provinces and encompasses 8 ethnic groups nationwide, provides high-quality multi-omics data resources for the localised application of artificial intelligence technology in the discovery of innovative drugs for metabolic diseases specific to the Chinese population, target validation and clinical translation, thereby establishing a solid data foundation. Emerging technologies systematically reduce the R&D costs of innovative drugs by significantly decreasing the time and financial investment required for key stages such as target discovery, compound screening and clinical trials. The systematic optimisation of the cost-effectiveness ratio further highlights the clinical and economic value of innovative drugs. Consequently, the weight assigned to innovation is expected to undergo a structural increase in future drug selection indicator systems.

The relatively low weighting of equity in access to medicines (5.07%) as another critical policy-aligned dimension reflects the need to optimise equitable resource allocation across populations within universal healthcare frameworks. To enhance equity, we recommend integrating health economics and bioethical frameworks into multidimensional equity assessment systems. First, disease burden weighting – such as disability-adjusted life years (DALYs) and societal health value parameters (e.g. prioritised treatment weights for children and working populations) – should be incorporated into cost-effectiveness analyses (CEA) to address limitations of single-threshold metrics like incremental cost-effectiveness ratio(ICER). Second, compensatory assessment mechanisms should be established for vulnerable groups (e.g. rare/geriatric diseases), permitting ICER threshold adjustments (20%−50% increase) (Bertram et al., 2021) and evaluating long-term cost-offset effects through Markov chain modelling (Aalabaf-Sabaghi, 2007).

**Figure 1.**
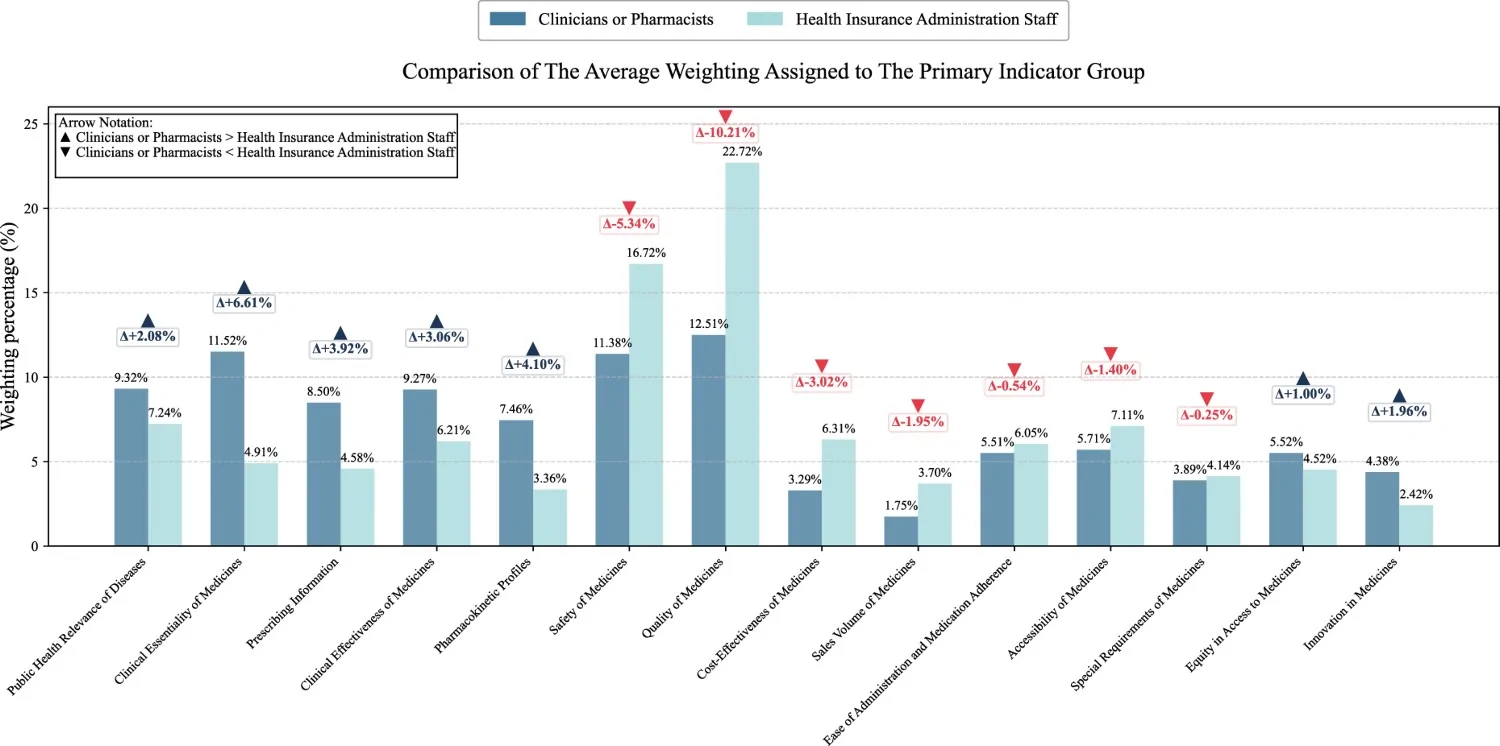
Comparison of the average weighting assigned by medical institution experts and health insurance administration experts to the primary indicator group. Note: $\chi^{2}\;(169)\;=\;182.00,\;\;p\;=\;.234.$ The critical value was obtained from the chi-square distribution table with α =   .05.

The statistical tests demonstrated no statistically significant differences in the weight allocation of 14 primary indicators between the Clinicians or Pharmacists group and the Health Insurance Administration Staff group (χ² = 182.00, df = 169, *p* = 0.234). However, comparative analysis of the average weight assignments by medical institution experts versus health insurance administration experts for the primary indicator groups (Figure 1) revealed several practically significant differential characteristics, revealing that payer experts prioritise safety of medicines (16.72%) and quality of medicines (22.72%), whereas clinical counterparts assign lower weights (11.38% and 12.51%, respectively). Conversely, clinicians emphasise clinical essentiality of medicines (11.52%), prescribing information (8.5%), and pharmacokinetic profiles (7.46%), with payer weights for these dimensions being markedly lower (4.91%, 4.58% and 3.36%). This divergence underscores the necessity for policymakers to balance drug safety/quality mandates with clinical demands for therapeutic transparency. Recommendations include mandating disclosure of manufacturing processes (e.g. generic drug bioequivalence evaluation reports) and real-world efficacy data through blockchain technology (Dong Jin, 2024) to ensure immutability and security.

This study's metabolic drug mini-HTA framework demonstrates innovative value but faces limitations: ⁣First⁣, its disease-specific weighting architecture (14 primary/42 secondary indicators) targets metabolic therapeutics’ unique clinical demands (e.g. chronicity-adjusted safety thresholds), limiting generalizability to other drug classes. Second, while the small expert panel (n = 7) ensured high domain expertise, it remains a limitation as it may restrict the broader representativeness of the consensus, and the Delphi-derived weights (Kendall’s W = 0.57) retain subjectivity risks where expert heterogeneity may skew prioritisation. ⁣Additionally⁣⁣, secondary indicators lack a direct quantitative scoring mechanism. In practical applications, it is necessary to further integrate quantitative tools (e.g. five-point Likert scales, interval scoring methods) or incorporate machine learning technologies. Finally, clinical utility requires retrospective validation (2020–2024 National Medical Insurance Catalog adjustments) and prospective piloting (provincial A/B testing). In conclusion, this study provides an indicator system and weight allocation scheme for the selection of metabolic drugs in China. However, before promoting the broad application of these research findings, in-depth analysis and necessary adjustments to the aforementioned limitations are required.

The metabolic drug accessibility assessment tool constructed in this study is based on a multidimensional weighted indicator system and ⁣⁣is planned to⁣⁣ integrate deep learning technologies in future iterations, enhancing the transparency, efficiency, precision and dynamic adaptability of ⁣⁣national drug inclusion decisions⁣⁣.

## Conclusion

This study established a hybrid AHP-Delphi-integrated mini-HTA decision framework through a quantifiable system of 14 primary and 42 secondary indicators, providing a standardised tool for evidence-based selection of metabolic medicines in the National Medical Insurance Catalog. The model demonstrated acceptable reliability with moderate consensus (Kendall’s W = 0.57; all CR < 0.1) while revealing critical policy balancing needs: safety-quality benchmarks dominated weighting allocation (cumulative 30.15%) versus underprioritized clinical innovation (3.23%). Stakeholder divergence emerged with payers emphasising safety (Δ+5.34%) and quality controls (Δ+10.21%), contrasting clinicians’ focus on therapeutic necessity (Δ+6.61%) and pharmacokinetic specificity (Δ+4.10%), highlighting the imperative for clinical-payer alignment in formulary design. Three implementation priorities are proposed: (1) technical development of regional disease spectrum-adaptive weighting algorithms, (2) data infrastructure for AI-driven real-world evidence aggregation and (3) policy innovation with subtype-specific ICER thresholds (e.g. diabetes/obesity). Despite certain limitations, the lightweight decision-making framework developed in this study not only optimises the dynamic adjustment mechanisms of China's National Medical Insurance Catalog but also its modular design features provide a replicable technical paradigm for implementing precision drug management in resource-limited regions.

## Supplementary Material

Supplementary Table S1.docx

Weights_FullData.xlsx

## Data Availability

Our data (results of the original questionnaire answered by experts) are not publicly available, and our ethics approval does not contain provisions to make them so.

## References

[CIT0001] Aalabaf-Sabaghi, M. (2007). Decision modelling for health economic evaluation. *Journal of Epidemiology and Community Health*, *61*(9), 839. 10.1136/jech.2007.059576

[CIT0002] Akins, R. B., Tolson, H., & Cole, B. R. (2005). Stability of response characteristics of a Delphi panel: Application of bootstrap data expansion. *BMC Medical Research Methodology*, *5*(1), 37. 10.1186/1471-2288-5-37 PMC1318466 16321161

[CIT0003] Anjana, R. M., Unnikrishnan, R., Deepa, M., Pradeepa, R., Tandon, N., Das, A. K., Joshi, S., Bajaj, S., Jabbar, P. K., Das, H. K., Kumar, A., Dhandhania, V. K., Bhansali, A., Rao, P. V., Desai, A., Kalra, S., Gupta, A., Lakshmy, R., Madhu, S. V., … Mohan, V. (2023). Metabolic non-communicable disease health report of India: The ICMR-INDIAB national cross-sectional study (ICMR-INDIAB-17). *The Lancet Diabetes & Endocrinology*, *11*(7), 474–489. 10.1016/s2213-8587(23)00119-5 37301218

[CIT0004] Bertram, M. Y., Lauer, J. A., Stenberg, K., & Edejer, T. T. T. (2021). Methods for the economic evaluation of health care interventions for priority setting in the health system: An update from WHO CHOICE. *International Journal of Health Policy and Management*, *10*(11), 673–677. 10.34172/ijhpm.2020.244 PMC9278384 33619929

[CIT0005] Center for Drug Evaluation, N. M. P. A. (2022). *2022 Annual drug review report* [*2022年度药品审评报告*]. https://www.cde.org.cn/main/news/viewInfoCommon/849b5a642142fc00738aff200077db11.

[CIT0006] Center for Drug Evaluation, N. M. P. A. (2023). *2023 Annual drug review report* [*2023年度药品评审报告*]. https://www.cde.org.cn/main/news/viewInfoCommon/b2e1b6d57a6c45c4af1c6a7b9bebbc2a.

[CIT0007] Center for Drug Evaluation, N. M. P. A. (2024). *2024 Annual drug review report* [*2024年度药品评审报告*]. 国家药品监督管理局. https://www.cde.org.cn/main/news/viewInfoCommon/54538c67b7e764fc51666567fc620241.

[CIT0008] China News Service. (2024, 10 September 2024). National Healthcare Security Administration elaborates on the “1+3+N” multi-level medical security system[国家医保局详解”1+3+N”多层次医疗保障体系]. https://www.chinanews.com.cn/gn/2024/09-10/10283388.shtml.

[CIT0009] Dolan, J. G., Isselhardt, B. J. Jr., & Cappuccio, J. D. (1989). The analytic hierarchy process in medical decision making: A tutorial. *Medical Decision Making*, *9*(1), 40–50. 10.1177/0272989(8900900108 2643019

[CIT0010] Fuchigami, A., Shigiyama, F., Kitazawa, T., Okada, Y., Ichijo, T., Higa, M., Hiyoshi, T., Inoue, I., Iso, K., Yoshii, H., Hirose, T., & Kumashiro, N. (2020). Efficacy of dapagliflozin versus sitagliptin on cardiometabolic risk factors in Japanese patients with type 2 diabetes: A prospective, randomized study (DIVERSITY-CVR). *Cardiovascular Diabetology*, *19*(1), 1. 10.1186/s12933-019-0977-z PMC6945792 31910850

[CIT0011] Geldsetzer, P., Manne-Goehler, J., Theilmann, M., Davies, J. I., Awasthi, A., Vollmer, S., Jaacks, L. M., Bärnighausen, T., & Atun, R. (2018). Diabetes and hypertension in India: A nationally representative study of 1.3 million adults. *JAMA Internal Medicine*, *178*(3), 363–372. 10.1001/jamainternmed.2017.8094 PMC5885928 29379964

[CIT0012] General Office of the State Council of the People's Republic of China. (2023). *Implementation opinions on strengthening routine supervision of healthcare fund usage* [*国务院办公厅关于加强医疗保障基金使用常态化监管的实施意见*]. (Guobanfa [2023] No. 17). The Central People's Government of the People's Republic of China​. Retrieved from https://www.gov.cn/zhengce/content/202305/content_6883811.htm.

[CIT0013] General Office of the State Council of the People's Republic of China. (2024). *Opinions on comprehensively deepening the reform of drug and medical device regulation and promoting the high-quality development of the pharmaceutical industry* [*国务院办公厅关于全面深化药品医疗器械监管改革促进医药产业高质量发展的意见*]. (Guobanfa [2024] No. 53). Retrieved from https://www.gov.cn/zhengce/content/202501/content_6996115.htm.

[CIT0014] Guest, G., Bunce, A., & Johnson, L. (2006). How many interviews are enough? An experiment with data saturation and variability. *Field methods*, *18*(1), 59–82. doi:10.1177/1525822X05279903

[CIT0015] International Diabetes Federation. (2025). *IDF diabetes atlas* (11th ed.). https://diabetesatlas.org/resources/idf-diabetes-atlas-2025/

[CIT0016] Jiajin, L., Tianxiao, H., Yujie, S., & Lu, Y. (2024). Development of HTA in the healthcare insurance systems of developed countries and its implications for China [HTA在发达国家医保体系中的发展及对我国的启示]. *China Healthcare Security*. (1), 100–110. https://cstj.cqvip.com/Qikan/Article/Detail?id=7111353552 (in chinese)

[CIT0017] Jiali, T., Li Qian, S. G., Yingqian, L., & Youli, H. (2023). Study on the construction and application of evaluation index system assessing quality of medicalinsurance service based on DIP with Delphi method and AHP [基于DIP的医保服务质量评价指标体系的构建与应用——基于德尔菲法和层次分析法]. *Chinese Journal of Health Policy*, *16*(2), 29–35. 10.3969/j.issn.1674-2982.2023.02.005 (in Chinese)

[CIT0018] Jianzhou, Y., Ruilin, D., & Rong, S. (2022). Enlightenment of WHO and some countries’ selection and withdrawal standards of essential medicine to China [WHO和部分国家基本药物遴选与退出标准对我国的启示]. *Chinese Journal of Health Policy*, *15*(4), 59–66. (in Chinese).

[CIT0019] Jin, D. (2024). Safeguarding national data security with blockchain technology [以区块链技术维护国家数据安全]. *China Cyberspace*, *08*, 37–40. https://kns.cnki.net/kcms2/article/abstract?v=gHWJqxSIlt2q1brs1zXnEAgOgCVSY4VrHI0qgm0Sx-og_91lEyTMsmlYhsdqkHzMkvtS6_vu6EeV3E6FGlgtKHxN1a2UuB3EufPf4VTi7g-tWfrzE-gRTDgwMJzsigPwB2srml9jrrLq5snfr8N2mnmLtNvrS6WhUuVZPIGH3GafDC_ExYJGmMZiyzN7eyBjXVO0_EoMsMg=&uniplatform=NZKPT&language=CHS (in Chinese)

[CIT0020] Kamya, P., Ozerov, I. V., Pun, F. W., Tretina, K., Fokina, T., Chen, S., Naumov, V., Long, X., Lin, S., Korzinkin, M., Polykovskiy, D., Aliper, A., Ren, F., & Zhavoronkov, A. (2024). PandaOmics: An AI-driven platform for therapeutic target and biomarker discovery. *Journal of Chemical Information and Modeling*, *64*(10), 3961–3969. 10.1021/acs.jcim.3c01619 PMC11134400 38404138

[CIT0021] Kenya National Bureau of Statistics. (2022). *Kenya demographic and health survey 2022* (DDI-KEN-KNBS-KDHS-2022-V001). https://statistics.knbs.or.ke/nada/index.php/catalog/128.

[CIT0022] Li, C., Hao, J., Zheng, Y., Wang, C., Yang, J., Wang, W., Zhang, K., Shao, C., Hui, W., Wang, J., Li, W., & Tang, Y. D. (2023). The changing landscape of drug clinical trials on cardiometabolic diseases in China, 2009-2021. *Diabetology & Metabolic Syndrome*, *15*(1), 66-1–66-13. 10.1186/s13098-023-01043-8 PMC10067219 37005689

[CIT0023] Liberatore, M. J., & Nydick, R. L. (2008). The analytic hierarchy process in medical and health care decision making: A literature review. *European Journal of Operational Research*, *189*(1), 194–207. 10.1016/j.ejor.2007.05.001

[CIT0024] Mao, W., Yip, C. W., & Chen, W. (2019). Complications of diabetes in China: Health system and economic implications. *BMC Public Health*, *19*(1), 269-1–269-11. 10.1186/s12889-019-6569-8 PMC6414024 30841928

[CIT0025] Ministry of Health and Family Welfare. (2022). *National List of Essential Medicines (NLEM) 2022*. G. o. India. https://mohfw.gov.in/?q=newshighlights-104.

[CIT0026] National Healthcare Security Administration (NHSA). (2020). *Interim measures for the administration of medicines covered by basic medical insurance* [*基本医疗保险用药管理暂行办法*]. Retrieved from https://www.nhsa.gov.cn/attach/0/bab0c20ab5604f69ab84ae22ab520274.pdf.

[CIT0027] National Health Commission of the People’s Republic of China. (2023). Adult hypertension dietary guidance (2023 edition) [成人高血压食养指南(2023年版)]. *​​​inical Education of General Practice​​*, *21*(6), 484–485,507. 10.13558/j.cnki.issn1672-3686.2023.006.002 (in chinese)

[CIT0028] National Health Commission of the PRC. (2020). Report on the nutrition and chronic diseases status of Chinese residents 2020 [中国居民营养与慢性病状况报告(2020年)]. *Acta Nutrimenta Sinica*, *42*(6), 521. in Chinese.

[CIT0029] (NHSA), N. H. S. A. (2024). *2024 work plan for adjusting the national medical insurance Catalog (NMIC) for basic medical insurance, work-related injury insurance, and maternity insurance” and related application guidelines* [*国家医疗保障局关于公布《2024年国家基本医疗保险、工伤保险和生育保险药品目录调整工作方案》及申报指南等文件的公告*]. (2024-04-00037). Retrieved from https://www.nhsa.gov.cn/art/2024/6/28/art_109_13084.html.

[CIT0030] O'Rourke, B., Oortwijn, W., & Schuller, T. (2020). The new definition of health technology assessment: A milestone in international collaboration. *International Journal of Technology Assessment in Health Care*, *36*(3), 187–190. 10.1017/s0266462320000215 32398176

[CIT0031] Saaty, R. W. (1987). The analytic hierarchy process – What it is and how it is used. *Mathematical Modelling*, *9*(3–5), 161–176. 10.1016/0270-0255(87)90473-8

[CIT0032] Saaty, T. L. (1977). A scaling method for priorities in hierarchical structures. *Journal of Mathematical Psychology*, *15*(3), 234–281. 10.1016/0022-2496(77)90033-5

[CIT0033] Social Security Network. (2025). *No application required for diabetes and special chronic diseases this year! 95% medical insurance reimbursement ratio, threshold fee abolished* [*今年!糖尿病慢特病无需申请,医保报销比例95%,门槛费取消*]. Retrieved from http://shebao.southmoney.com/yiliao/202503/787802.html.

[CIT0034] Temu, T. M., Macharia, P., Mtui, J., Mwangi, M., Ngungi, P. W., Wanjalla, C., Bloomfield, G. S., Farquhar, C., Nyanjau, L., Gathecha, G. K., & Kibachio, J. (2021). Obesity and risk for hypertension and diabetes among Kenyan adults: Results from a national survey. *Medicine (Baltimore)*, *100*(40), e27484. 10.1097/md.0000000000027484 PMC8500651 34622879

[CIT0035] Ting, Wang. (2022). Reflections on the application of multi-criteria decision analysis (MCDA) in medical insurance catalog review [多准则决策分析(MCDA)在医保目录评审中应用的思考]. *Consumption Guide* (18), 32–35. 10.12229/j.issn.1672-5719.2022.18.009 (in Chinese)

[CIT0036] Vaidya, O. S., & Sushil, K. (2006). Analytic hierarchy process: An overview of applications. *European Journal of Operational Research*, *169*(1), 1–29. 10.1016/j.ejor.2004.04.028

[CIT0037] Writing Group of Chinese Guidelines for the Management of Hypertension, H. L. o. C.,: Hypertension Branch of China International Exchange and Promotive Association for Medical and Health Care, Geriatric Hypertension Branch of Chinese Geriatric Society, Hypertension Branch of China Aging Health Association, Chinese Stroke Association (Wang, J. G. (2024). 2024 Chinese guidelines for the management of hypertension [中国高血压防治指南(2024年修订版)]. *Chinese Journal of Hypertension*, *32*(7), 603–700. https://qikan.cqvip.com/Qikan/Article/Detail?id=7112805540

[CIT0038] World Health Organization. (2023a). *Kenya essential medicines list 2023 (English)*. G. o. Kenya. https://www.who.int/publications/m/item/kenya–essential-medicines-list-2023-(english).

[CIT0039] World Health Organization. (2023b). *The selection and use of essential medicines 2023. Executive summary of the report of the 24th WHO Expert Committee on selection and use of essential medicines.* (WHO/MHP/HPS/EML/2023.02). https://www.who.int/publications/i/item/WHO-MHP-HPS-EML-2023.01.

[CIT0040] Xu, Z., Ren, F., Wang, P., Cao, J., Tan, C., Ma, D., Zhao, L., Dai, J., Ding, Y., Fang, H., Li, H., Liu, H., Luo, F., Meng, Y., Pan, P., Xiang, P., Xiao, Z., Rao, S., Satler, C., … Zhavoronkov, A. (2025). A generative AI-discovered TNIK inhibitor for idiopathic pulmonary fibrosis: A randomized phase 2a trial. *Nature Medicine*, 31(8), 2602–2610. 10.1038/s41591-025-03743-2 PMC12353801 40461817

[CIT0041] Yan, Y., Mi, T., & Jiangjiang, H. (2023). A Study on the multidimensional decision framework for health technology assessment from the perspective of adjusting the medical insurance drug catalog [医保药品目录调整视角下的卫生技术评估多维度决策框架研究]. *Chinese Health Economics*, *42*(11), 28–32. https://kns.cnki.net/kcms2/article/abstract?v=gHWJqxSIlt3joE7ykc7Cz22r6l5b18CcG74QA31AZk-VQcq7iTlH0oxi65izI_wbTk21PdrMZnmsmulrPGMEGquGXVImqiaZXik_dCO9iaQapGyDJI-emqRg-vQ8sxsWhcE9dS6IhRgE5WhgVCuN9a1Wtq6KQu1hympi-N9nkRYp7odbyeC3br9gAUFczPWXFCqgTcXXPZA=&uniplatform=NZKPT&language=CHS (in Chinese)

[CIT0042] Ye, Y., Liu, X., Wu, N., Han, Y., Wang, J., Yu, Y., & Chen, Q. (2021). Efficacy and safety of berberine alone for several metabolic disorders: a systematic review and meta-analysis of randomized clinical trials. *Frontiers in Pharmacology*, *12*, 653887. 10.3389/fphar.2021.653887 PMC8107691 33981233

[CIT0043] Yunxiang, Z. H. O. N. G., Lin, Y. I. N., Haipeng, T. A. N. G., Aihong, W. A. N. G., Qianwen, Z. H. A. N. G., & Song, J. I. A. N. G. (2025). Medical insurance drug admission: (colon)A comparison of Mini – HTA and RHTA [医保药品准入: (colon)Mini-HTA与RHTA适用性比较]. *​China Pharmaceuticals*, *34*(08), 21–25. https://kns.cnki.net/kcms2/article/abstract?v=gHWJqxSIlt0XeRUAUuFNW1Wda2Cz43ZbZ4U4bMx4_qBDOuL0ZsrC333k1hD5vQ4oX2YuGo2Tu2ZXVYq_P4y7ZcJ5Ur1LONN51NMvyc6WPKcN8UmqMzDTiJ3pdbtBBQpSLMXEwXpc6BtFwn01u8KXUiZEYcEm6S4KuEnZkPBf4RFnZaP9DmKHG_dtXXpjABQ2VO0ocZ5nb64=&uniplatform=NZKPT&language=CHS (in chinese)

[CIT0044] Zhengxiang, L., Yu, Z., Lingli, Z., & Rong, D. (2022). Drug selection guideline for medical institutions [医疗机构药品遴选指南]. *China Pharmacy*, *33*(07), 769–776. https://link.cnki.net/urlid/50.1055.r.20220318.1350.002 (in Chinese)

[CIT0045] Zhigang, Z., zhanjun, D., & Jianping, L. (2023). A quick guideline for drug evaluation and selection in Chinese medical institutions (the Second Edition) [中国医疗机构药品评价与遴选快速指南(第二版)]. *Herald of Medicine*, *42*(4), 447–456. (in Chinese)

