# Supplementary material for: Integrated AHP-Delphi and Mini-HTA for selection of metabolic drugs in China’s national medical insurance catalog: a framework towards quantitative dynamic adjustment
Source: J Pharm Policy Pract. 2026 Sep 21;19(1):2701914. doi: 10.1080/20523211.2026.2701914 (PMC13600306; doi:10.1080/20523211.2026.2701914)
Supplement: Supplementary file 1 [file JPPP_A_2701914_SM7048.docx]

| PRIMARY INDICATORS | SECONDARY INDICATORS |
| --- | --- |
| 1. Public Health Relevance of Diseases | 1.1 The Epidemiological Information of this Disease in China (including Total Affected Population, Susceptible Populations, Prevalence Rate, Mortality Rate, Comorbidity Incidence Rate and others) |
| 2. Clinical Essentiality of Medicines | 2.1 Medicines for Rare Diseases |
|  | 2.2 Use of Medicines in Specific Populations |
|  | 2.3 Historical Data on Medication Use |
|  | 2.4 Limitations of Existing Medicines in Clinical Therapeutic Use |
| 3. Prescribing Information | 3.1 Target Population, Dosing Regimen, Treatment Duration, Adverse Drug Reactions, and Pharmacokinetics |
|  | 3.2 Is the Completeness of Additional Information (Pharmaceutical Properties, Drug Interactions, Drug Overdose, Specifications, and Related Parameters) |
| 4. Clinical Effectiveness of Medicines | 4.1 Average Score of Inclusion Status Across Pharmacopoeias: Score for Chinese Pharmacopoeia Inclusion, Score for Non-Pharmacopoeial National Drug Standards Inclusion, Score for International Pharmacopoeia Inclusion Frequency |
|  | 4.2 Guideline Recommendations: Chinese Mainland Guideline Recommendation Score; Southeast Asian Region Guideline Recommendation Frequency Score; Other High-Prevalence Regions for Metabolic Diseases Guidelisne Recommendation Frequency Score |
|  | 4.3 Evidence-Based Medicine Research Findings: Comparative Impact of Pharmacotherapy Versus No Intervention, Versus Comparator Drugs, Versus Other Therapeutic Modalities on Patient Clinical Outcomes: Systematic Review/Meta-Analysis (Network) Ranking Score; Randomized Controlled Trials Ranking Score; Cohort Studies or Case-Control Studies Ranking Score; Pathological Reports or Case Observation Studies Ranking Score |
|  | 4.4 Is the Inclusion of Indication-Related Diseases in Clinical Pathways |
|  | 4.5 Is it an FDC |
|  | 4.6 Healthcare Professionals' High Acceptance Level |
|  | 4.7 Broad Applicability: Substantial Evidence Demonstrates Stable Therapeutic Efficacy in Various Settings (e.g., Different Healthcare Levels) |
| 5. Pharmacokinetic Profiles | 5.1 Are the Absorption, Distribution, Metabolism, Excretion, and Bioavailability of The Medicine Satisfactory |
|  | 5.2 Is it Nonlinear Pharmacokinetics |
| 6. Safety of Medicines | 6.1 Incidence of Adverse Reactions, Severity (Impact on Patient Quality of Life, Disease Prognosis, and Economic Burden) |
|  | 6.2 Comparison of Adverse Reactions with Comparator Drugs |
|  | 6.3 Reporting Frequency of Adverse Reaction Information (Product Labeling Warnings and Safety Risk Notifications/Alerts/Communications in China, US, Europe, and Japan) |
|  | 6.4 Contraindications and Drug Interactions |
|  | 6.5 Development Status of the Pharmacovigilance System |
|  | 6.6 Evidence and Quality Levels of Safety Studies for Candidate Medicines: Systematic Reviews and Meta-Analyses; Randomized Controlled Trials; Other Types of Clinical Controlled Studies |
|  | 6.7 Applicability of Candidate Medicines in Special Populations |
| 7. Quality of Medicines | 7.1 Quality Status Scores of Manufacturing Enterprises |
|  | 7.2 Whether the Drug is an Innovator Drug; If Generic Drugs have Passed Pharmaceutical Equivalence Evaluation |
|  | 7.3 Export and Sales Status of Domestic Drugs (Registration, Application, and Sales Status in Foreign Countries) |
|  | 7.4 Development of Quality Standards Control System |
|  | 7.5 Quality Stability: Compliance with Internationally Recognized Quality Standards Acknowledged by Pharmaceutical Regulatory Authorities, including in Production, Storage, Transportation, and Use |
| 8. Cost-Effectiveness of Medicines | 8.1 Cost-Effectiveness Comparisons with Comparator Drugs or Other Therapeutic Modalities: Unit Price; Daily Cost; Full Course Cost |
|  | 8.2 Cost-Effectiveness Literature Research Findings Ranking Score |
| 9. Sales Volume of Medicines | 9.1 Sales Volume of Candidate Medicines |
| 10. Ease of Administration and Medication Adherence | 10.1 Frequency of Medication Use |
|  | 10.2 Appropriate Packaging, Facilitated Accessibility, and Adaptability to Healthcare Institutions of All Scales |
|  | 10.3 Route of Administration (Oral, Injection, Topical, etc.) |
|  | 10.4 Whether the Taste of Medication, Side Effects, etc. Align with Patient Preferences and Needs |
|  | 10.5 Storage Conditions of Medicines |
|  | 10.6 Other Information on Adherence-Related Factors of Candidate Medicines |
| 11. Accessibility of Medicines | 11.1 Marketing Status of Medicines; Marketing Status of Generic Drugs; Whether the Product has been Approved by Local Pharmaceutical Regulatory Authorities |
|  | 11.2 Availability of Candidate Medicines (Manifested by Shortage Level) |
| 12. Special Requirements of Medicines | 12.1 Whether the Use of the Product requires Professional Training, Diagnosis, Management, Monitoring, or Other Skills |
| 13. Equity in Access to Medicines | 13.1 Whether Inclusion would lead to Diversion of Limited Healthcare Funds from a Larger Patient Population or Higher-Priority Public Health Products; whether it Pertains to a High-Cost Medicine with a Small Patient Population, where Non-Inclusion may Result in Increased Long-Term Treatment or Care Costs |
| 14. Innovation in Medicines | 14.1 Whether Candidate Medicines can address Gaps in the Therapeutic Formulary |
|  | 14.2 Whether Candidate Medicines demonstrate Innovativeness in the Scope of Therapeutic Indications |
|  | 14.3 Whether Candidate Medicines demonstrate Innovativeness in the Context of Pharmacological Mechanisms and Pharmaceutical Formulations |

**Note. Detailed Description of Primary Indicators and Their Corresponding Secondary Indicators**

1. **Public Health Relevance of Diseases:** This indicator aims to prioritize the assessment of the overall health burden and intervention urgency of metabolic diseases on the Chinese population from a macro-policy perspective. Its core is to quantify disease impact through secondary indicators (1.1 Epidemiological Information of the Disease in China), specifically including key data such as the total number of cases, characteristics of susceptible populations, prevalence rate, mortality rate, and comorbidity incidence rate. This design fully considers the typical characteristics of metabolic drugs: such diseases usually have a large affected population, require long-term medication due to prolonged course, are prone to cause serious complications such as cardiovascular and cerebrovascular diseases, and involve complex comorbidity management. Therefore, epidemiological data not only directly reflects the public health importance of the disease but also provides an objective basis for catalog selection, ensuring that limited resources are prioritized for metabolic disease drugs that cover a wide population, have a high complication burden, and address high unmet clinical needs, thereby maximizing the protection benefits.
2. **Clinical Essentiality of Medicines:** This indicator aims to assess the irreplaceability of metabolic drugs for different diseases and populations from the perspective of clinical demand. It is systematically measured through four secondary indicators: 2.1 Drugs for Rare Diseases (focusing on the accessibility and life-sustaining value of drugs for rare metabolic diseases), 2.2 Medication for Specific Populations (emphasizing dosage adjustment and safety needs for special groups such as children, the elderly, and individuals with hepatic or renal insufficiency), 2.3 Historical Data on Drug Use (analyzing the stability of long-term medication efficacy and patterns of real-world adverse drug reactions), and 2.4 Clinical Limitations of Existing Drugs (identifying unmet needs in current therapies regarding target attainment rates for blood glucose/lipids/blood pressure control, target organ protection, etc.). This design closely aligns with the characteristics of metabolic drugs, which involve "long-term medication use, complex complications, and strong population heterogeneity," and emphasizes the personalized treatment value of drugs for special metabolic populations (e.g., adolescents with diabetes, elderly patients with comorbidities). By quantifying clinical essentiality, it ensures that the formulary prioritizes the inclusion of high-value drugs that can fill therapeutic gaps, improve long-term prognoses, and meet the needs of specific metabolic populations at various life stages, thereby optimizing the clinical output efficiency of limited medical insurance resources.
3. **Prescribing Information:** This indicator aims to systematically evaluate the completeness and clinical applicability of prescribing information for metabolic drugs, providing a structured basis for core medication decision-making. It is broken down into two secondary indicators: 3.1 Target Population, Dosage Regimen, Treatment Cycle, Adverse Reactions, and Pharmacokinetics, focusing on the dose adjustment needs for specific metabolic populations (e.g., patients with hepatic or renal insufficiency, elderly patients with comorbidities), long-term medication safety, and in vivo metabolic characteristics (e.g., absorption rate and distribution half-life of oral hypoglycemic drugs); 3.2 Completeness of Supplementary Information (covering pharmaceutical properties, drug interactions, overdose management, specifications, and related parameters), emphasizing the evaluation of drug stability in complex metabolic environments (e.g., insulin storage conditions), risks of drug combinations (e.g., interactions between statins and hypoglycemic drugs), and strategies for addressing medication errors. This design closely aligns with the characteristics of metabolic drugs, which involve "long-term medication use, complex pharmacokinetics, and safety sensitivity," and highlights the supportive role of prescribing information for personalized treatment. Through a comprehensive review of prescription data, it ensures that the formulary prioritizes the inclusion of drugs with transparent information, standardized use, and controllable risks, thereby reducing safety hazards during long-term medication use and optimizing the investment efficiency of medical insurance funds in chronic disease management.
4. **Clinical Effectiveness of Medicines:** This indicator aims to systematically evaluate the clinical benefits and practical therapeutic value of metabolic drugs based on evidence-based medical evidence, providing multidimensional empirical support for core medication decision-making. It is structurally expanded through seven secondary indicators: 4.1 Pharmacopoeia Inclusion Status Average Score (quantifying the frequency of drug inclusion in the Chinese Pharmacopoeia, non-pharmacopoeia national standards, and international pharmacopoeias, reflecting basic recognition); 4.2 Guideline Recommendations (counting the recommendation frequency in clinical guidelines from regions with high metabolic disease burdens such as mainland China and Southeast Asia, highlighting the characteristics of metabolic diseases requiring long-term management and significant regional variation); 4.3 Evidence-Based Medical Research Results (comprehensively evaluating the impact of drugs versus no intervention/comparator drugs/other treatment modalities on clinical outcomes through evidence grading of systematic reviews/meta-analyses, randomized controlled trials, cohort studies, etc.); 4.4 Whether the Indication-Related Disease is Included in Clinical Pathways; 4.5 Whether it is a Fixed-Dose Combination (FDC); 4.6 High Acceptance Among Healthcare Professionals; 4.7 Broad Applicability (based on evidence of treatment stability across multiple scenarios). This design closely aligns with the characteristics of metabolic drugs, which "require long-term medication use, have complex complications, and need multi-level verification of efficacy evidence," emphasizing the consistency of drug efficacy and clinical operability in different healthcare environments. By systematically assessing the chain of evidence for clinical effectiveness, it ensures that the formulary prioritizes the inclusion of metabolic drugs that are highly recommended by guidelines, have high evidence-based grades, are highly accepted clinically, and are applicable in broad scenarios, thereby optimizing the input-output efficiency of medical insurance funds in the long-term management of chronic diseases.
5. **Pharmacokinetic Profiles:** This indicator aims to systematically evaluate the dynamic processes and exposure characteristics of metabolic drugs in the human body, providing key scientific evidence on drug in vivo behavior for selection. It is meticulously examined through two secondary indicators: 5.1 Whether the drug's absorption, distribution, metabolism, excretion, and bioavailability are satisfactory, with particular focus on the absorption stability, changes in volume of distribution, risks of metabolic enzyme inhibition/induction, and potential accumulation due to abnormal excretion pathways in special populations (e.g., patients with hepatic or renal insufficiency, elderly patients with comorbidities) for metabolic drugs such as oral hypoglycemic agents and lipid-lowering drugs; 5.2 Whether it exhibits nonlinear pharmacokinetics, identifying drugs with a non-proportional dose-exposure relationship (e.g., certain antihypertensive or anticoagulant drugs with a narrow therapeutic window), which are more prone to efficacy or safety mutations triggered by minor dose adjustments during long-term use. This design closely aligns with the characteristics of metabolic drugs, which involve "long-term medication use, highly variable patient physiological status, and complex drug interactions," emphasizing the decisive role of pharmacokinetic profiles in personalized dose adjustment and long-term safety. By comprehensively assessing ADME properties and linearity characteristics, it ensures that the formulary prioritizes the inclusion of drugs with stable pharmacokinetic behavior, strong predictability, and low safety risks, thereby reducing treatment failures or adverse reactions in patients due to physiological changes or drug combinations, and optimizing the long-term benefit-risk balance of medical insurance funds in chronic disease management.
6. **Safety of Medicines:** This indicator aims to systematically evaluate the safety risks and tolerability profiles of metabolic drugs in real-world clinical applications, providing comprehensive evidence on drug safety for medical insurance catalog selection. It is structurally expanded through seven secondary indicators: 6.1 Incidence and Severity of Adverse Reactions (focusing on the impact on patients' quality of life, disease prognosis, and economic burden, particularly addressing potential disorders of glucose and lipid metabolism, hepatic and renal dysfunction, and daily life interference caused by long-term use of metabolic drugs); 6.2 Comparison of Adverse Reactions with Control Drugs (identifying relative safety advantages); 6.3 Frequency of Domestic and International Pharmacovigilance Information Reports (covering product label warnings and safety risk notifications from China, the U.S., Europe, and Japan); 6.4 Contraindications and Drug Interactions (assessing combination therapy risks); 6.5 Development Status of Pharmacovigilance Systems; 6.6 Evidence and Quality Level of Safety Studies for Candidate Drugs (including systematic reviews/meta-analyses, randomized controlled trials, and other clinical controlled studies); 6.7 Applicability of Candidate Drugs in Special Populations. This design closely aligns with the characteristics of metabolic drugs, which are "often used for chronic disease management, involve special patient populations (e.g., those with hepatic or renal insufficiency, elderly patients with comorbidities), and require long-term medication," emphasizing the identification of potential risks associated with prolonged use through multidimensional safety data. By systematically evaluating the spectrum of adverse reactions and the strength of evidence, it ensures that the catalog prioritizes the inclusion of drugs with well-defined safety profiles, controllable risks, and strong applicability in special populations, thereby optimizing the long-term treatment safety for chronic disease patients and the sustainable utilization of medical insurance funds.
7. **Quality of Medicines:** This indicator aims to systematically evaluate the quality reliability and standards compliance of metabolic drugs throughout their entire lifecycle from production to use, providing critical quality evidence support for medical insurance catalog selection. It is structurally expanded through five secondary indicators: 7.1 Manufacturer Quality Status Score (focusing on the pharmaceutical manufacturer's GMP compliance, production consistency, and failed sampling inspection rate, reflecting the enterprise's capability to consistently supply high-quality metabolic drugs such as insulin and oral hypoglycemic agents); 7.2 Whether the Drug is an Innovative Drug, and if it is a Generic Drug, Whether it has Passed Drug Equivalence Evaluation (ensuring that generic hypoglycemic and lipid-lowering drugs are consistent with the originator drug in terms of bioavailability, efficacy stability, and safety, meeting the reliability requirements for long-term medication use); 7.3 Export and Sales Status of Domestic Drugs (using registration, application, and sales data from strictly regulated regions such as Europe and America to indirectly verify international quality recognition, particularly focusing on stability validation of metabolic drugs under high temperature and high humidity conditions); 7.4 Development of Quality Standard Control Systems (assessing whether enterprises adopt stricter internal control standards and advanced analytical technologies to control the risk of degradation products and impurities that may arise during the production of metabolic drugs); 7.5 Quality Stability (focusing on whether the drug complies with internationally recognized quality standards approved by regulatory authorities during production, storage, transportation, and use, particularly the thermal stability of biological products such as insulin, the dissolution of oral drugs, and content stability within the validity period). This design closely aligns with the characteristics of metabolic drugs, which involve "long medication cycles, sensitivity to storage conditions, and high requirements for bioavailability," emphasizing the safeguarding role of full lifecycle quality control on efficacy and safety. By systematically evaluating the quality dimension, it ensures that the catalog prioritizes the inclusion of drugs with mature production processes, stable quality, high international recognition, and confirmed generic drug equivalence, thereby reducing efficacy deficiencies or safety risks caused by quality fluctuations and optimizing the long-term value of medical insurance funds in chronic disease management.
8. **Cost-Effectiveness of Medicines:** This indicator aims to systematically evaluate the balance between the economic value and clinical benefits of metabolic drugs, providing critical pharmacoeconomic evidence to support medical insurance catalog selection. It is structurally expanded through two secondary indicators: 8.1 Cost-Effectiveness Comparison with Control Drugs or Other Treatment Modalities (comprehensively considering drug unit price, average daily treatment cost, and total course expense, with a focus on analyzing the cumulative economic burden versus health output ratio of metabolic drugs such as insulin and oral hypoglycemic agents during long-term use); 8.2 Ranking Score of Cost-Effectiveness Literature Research Results (quantitatively scoring and ranking the cost-effectiveness ratios of different metabolic drugs based on domestic and international pharmacoeconomic research evidence, through meta-analyses and modeling studies). This design closely aligns with the characteristics of metabolic drugs, which involve "prolonged medication cycles, the need for lifelong patient treatment, and significant healthcare cost burdens," emphasizing the critical role of pharmacoeconomic evaluation in optimizing the allocation of medical insurance funds and alleviating the long-term economic pressure on patients. By systematically assessing the cost-effectiveness dimension, it ensures that the catalog prioritizes the inclusion of drugs with clear clinical benefits, excellent economic efficiency, and high cost-effectiveness for long-term therapy, thereby optimizing the input efficiency and sustainability of medical insurance funds in chronic disease management.
9. **Sales Volume of Medicines:** This indicator aims to systematically evaluate the market scale and patient acceptance of metabolic drugs in real-world clinical use, providing key practical evidence on drug market performance and demand matching for medical insurance catalog selection. Its core is meticulously examined through the secondary indicator 9.1 Candidate Drug Sales Volume, focusing on the prescription volume, sales amount, and market coverage of metabolic drugs such as oral hypoglycemic agents, insulin, and lipid-lowering drugs in medical institutions and retail terminals, reflecting the frequency of drug use in real medical environments and the recognition by doctors and patients. This design closely aligns with the characteristics of metabolic drugs, which involve "long-term medication needs, high patient dependence, and stable market usage," emphasizing the important reference value of sales volume data for the clinical applicability of drugs, the stability of production supply, and the predictability of medical insurance fund expenditures. By systematically evaluating the sales dimension, it ensures that the catalog prioritizes the inclusion of drugs with high clinical recognition, strong supply guarantee, and good patient medication adherence, thereby optimizing the practicality and sustainability of the medical insurance catalog.
10. **Ease of Administration and Medication Adherence:** This indicator aims to systematically evaluate the operational convenience of metabolic drugs in practical use and patients' long-term treatment adherence, providing critical evidence on drug practicality and patient acceptability for medical insurance catalog selection. It is structurally expanded through six secondary indicators: 10.1 Frequency of Medication Use (focusing on the impact of daily or weekly dosing frequency on long-term treatment adherence in chronic disease patients); 10.2 Appropriate Packaging, Facilitated Accessibility, and Adaptability to Healthcare Institutions of All Scales (assessing whether drug packaging facilitates opening, carrying, and dose identification for elderly patients or those with limited mobility); 10.3 Route of Administration (Oral, Injection, Topical, etc.) (comparing the convenience and patient preferences of different administration methods such as oral, injection, and topical in practical use); 10.4 Whether the Taste of Medication, Side Effects, etc. Align with Patient Preferences and Needs (particularly focusing on the tolerance of children and elderly patients to taste, swallowing difficulty, and adverse reactions); 10.5 Storage Conditions of Medicines (analyzing the stringency of drug requirements for temperature, humidity, and other environmental conditions, and their feasibility in household or primary healthcare settings); 10.6 Other Information on Adherence-Related Factors of Candidate Medicines (such as supporting measures including medication reminder devices and patient education materials). This design closely aligns with the characteristics of metabolic drugs, which involve "long medication cycles, high patient self-management demands, and direct impact of long-term adherence on treatment efficacy," emphasizing the decisive role of administration ease and patient acceptance in treatment success. By systematically evaluating the adherence dimension, it ensures that the catalog prioritizes the inclusion of drugs that are easy to use, highly accepted by patients, and conducive to long-term treatment adherence, thereby improving chronic disease management outcomes and optimizing the input-output efficiency of medical insurance funds in real-world settings.
11. **Accessibility of Medicines:** This indicator aims to systematically evaluate the availability and supply stability of metabolic drugs in the healthcare market, providing critical evidence on the marketing status and continuous supply capacity of drugs for medical insurance catalog selection. It is meticulously examined through two secondary indicators: 11.1 Marketing Status of Medicines (focusing on whether metabolic drugs and their generic versions have obtained market approval from local regulatory authorities such as China's National Medical Products Administration (NMPA), ensuring drugs possess legal market access qualifications); 11.2 Supply Status of Candidate Medicines (Manifested by Shortage Level) (emphasizing the analysis of stability in the production and distribution processes of metabolic drugs such as insulin and oral hypoglycemic agents, with particular attention to potential shortage risks caused by complex production processes, fluctuations in raw material supply, or sudden surges in market demand). This design closely aligns with the characteristics of metabolic drugs, which involve "long-term medication needs for patients, where treatment interruption may lead to severe health risks such as loss of glycemic/lipid control," emphasizing the decisive role of drug accessibility in ensuring treatment continuity and medication safety. By systematically evaluating marketing status and supply stability, it ensures that the catalog prioritizes the inclusion of drugs that are already approved for marketing, have strong supply guarantees, and low shortage risks, thereby reducing treatment interruptions or disease recurrence due to drug shortages and optimizing the sustainability of medical insurance funds in the long-term management of chronic diseases.
12. **Special Requirements of Medicines:** This indicator aims to systematically evaluate the special dependence of metabolic drugs on professional skills and management during clinical use, providing key evidence on medication safety and operational standardization for medical insurance catalog selection. Its core is meticulously examined through the secondary indicator 12.1: whether product use requires professional training, diagnosis, management, monitoring, or other skills, focusing on the assessment of specialized competency requirements such as insulin injection techniques, operation of blood glucose/lipid monitoring equipment, dose adjustment algorithms, adverse reaction identification, and emergency handling. This design closely aligns with the characteristics of metabolic drugs, which involve "complex medication regimens, high patient self-management demands, and improper operation easily leading to loss of efficacy or safety risks," emphasizing the decisive role of professional skill support in ensuring treatment effectiveness and medication safety. By systematically evaluating special skill requirements, it ensures that the catalog prioritizes the inclusion of drugs with clear operational specifications, well-established training systems, and accessible medical support, thereby reducing treatment failures or adverse events due to improper use and optimizing risk control and long-term benefits of medical insurance funds in the refined management of chronic diseases.
13. **Equity in Access to Medicines:** This indicator aims to systematically evaluate the impact of including metabolic drugs in the medical insurance catalog on the equity of healthcare resource allocation and long-term benefits, providing key evidence for fund optimization and risk balance in the selection process. Its core is meticulously examined through the secondary indicator 13.1: whether inclusion leads to the diversion of limited medical funds from larger patient populations or higher-priority public health products, focusing on the risk that metabolic drugs such as insulin and novel hypoglycemic agents may crowd out funding for essential chronic disease medications; it also assesses whether the drug is a high-cost, small-patient-population medication, and whether treatment discontinuation due to non-inclusion would lead to increased long-term care costs such as diabetic complications, cardiovascular and cerebrovascular events. This design closely aligns with the characteristics of metabolic drugs, which involve "large differences in patient population size, high costs of some highly innovative drugs, and treatment continuity directly impacting long-term prognosis," emphasizing the decisive role of equity assessment in optimizing fund utilization efficiency and safeguarding patient rights. By systematically analyzing fund diversion effects and long-term cost impacts, it ensures that the catalog prioritizes the inclusion of drugs that both meet special clinical needs and do not compromise population equity, thereby optimizing the strategic allocation efficiency of medical insurance funds in the stratified management of metabolic diseases.
14. **Innovation in Medicines:** This indicator aims to systematically evaluate the breakthrough value of metabolic drugs in terms of therapeutic strategy, mechanism of action, and formulation design, providing key evidence on drug innovation and therapeutic advancement for medical insurance catalog selection. It is meticulously examined through three secondary indicators: 14.1 Whether Candidate Medicines can address Gaps in the Therapeutic Formulary (focusing on whether the drug addresses unmet clinical needs in the field of metabolic diseases, such as the additional protective effects of novel hypoglycemic agents against cardio-renal complications); 14.2 Whether Candidate Medicines demonstrate Innovativeness in the Scope of Therapeutic Indications (assessing whether the drug expands traditional treatment boundaries, such as the multi-indication breakthrough of GLP-1 receptor agonists from hypoglycemia to weight loss and cardiovascular benefits); 14.3 Whether Candidate Medicines demonstrate Innovativeness in the Context of Pharmacological Mechanisms and Pharmaceutical Formulations (analyzing whether the drug adopts new targets, new pathways, or innovative formulation technologies, such as dual-target agonists, oral peptide formulations, and other cutting-edge interventions targeting the complex pathophysiology of metabolic diseases). This design closely aligns with the characteristics of metabolic drugs, which involve a "complex disease spectrum, diverse pathological mechanisms, and significant individual patient differences," emphasizing the decisive role of innovation in enhancing treatment precision and breaking clinical bottlenecks. By systematically evaluating the innovation dimension, it ensures that the catalog prioritizes the inclusion of drugs with breakthrough therapeutic value, capable of addressing clinical pain points and representing future treatment directions, thereby optimizing the strategic value and long-term benefits of medical insurance funds in the innovative transformation of metabolic disease management.
